# Limitation of Optical Enhancement in Ultra-thin Solar Cells Imposed by Contact Selectivity

**DOI:** 10.1038/s41598-018-27155-0

**Published:** 2018-06-11

**Authors:** Raisul Islam, Krishna Saraswat

**Affiliations:** Department of Electrical Engineering, Stanford University, 420 Via Palou Mall, Stanford, CA 94305 USA

## Abstract

Ultra-thin crystalline silicon (c-Si) solar cell suffers both from poor light absorption and minority carrier recombination at the contacts resulting in low contact selectivity. Yet most of the research focuses on improving the light absorption by introducing novel light trapping technique. Our work shows that for ultra-thin absorber, the benefit of optical enhancement is limited by low contact selectivity. Using simulation we observe that performance enhancement from light trapping starts to saturate as the absorber scales down because of the increase in probability of the photo-generated carriers to recombine at the metal contact. Therefore, improving the carrier selectivity of the contacts, which reduces the recombination at contacts, is important to improve the performance of the solar cell beyond what is possible by enhancing light absorption only. The impact of improving contact selectivity increases as the absorber thickness scales below 20 micrometer (*μ*m). Light trapping provides better light management and improving contact selectivity provides better photo-generated carrier management. When better light management increases the number of photo-generated carriers, better carrier management is a useful optimization knob to achieve the efficiency close to the thermodynamic limit. Our work explores a design trade-off in detail which is often overlooked by the research community.

## Introduction

Ultra-thin c-Si solar cells (having absorber layer of sub-10 *μ*m) offer versatile benefit for energy harvesting. This technology has the potential to provide better stability and higher efficiency at low cost^1,2^. Through layer transferring, ultra-thin c-Si photovoltaics (PV) provides a path towards shrinking the material consumption cost by allowing multiple utilization of the substrate^3^, which currently accounts for 40% of the module cost^4^. Thin absorber material is essential for flexible photovoltaics, creating the opportunity for photovoltaic energy harvesting in wireless sensor networks used in Internet-of-Things (IoT) application^5^. While the common flexible PV technologies (including the traditional thin-film technologies) are cheap and scalable, they suffer from long term stability and their potential for higher efficiency is limited, which is an important factor for energy harvesting in area constraint system like IoT sensors. Ultra-thin c-Si solar cell, if integrated with perovskite cells as tandems can promise >30% efficiency for a flexible PV system^6^. Ultra-thin c-Si PV technology can also harvest energy through on-chip embedded solar cells^7^ or 3-D integration of solar cells onto chips^8^. This extends the usability of IoT sensor chips in harsh and natural environment for agricultural or environmental monitoring, where batteries may not be a sustainable energy harvesting source.

Unfortunately, silicon is not a strong absorber of sunlight despite holding more than 70% share of the overall PV industry. Therefore, scaling of the thickness of c-Si solar cell while ensuring the same or even better efficiency has been a significant research problem to this date^4^. The main challenge of scaling down the thickness of the absorber of c-Si solar cell is to make sure the solar spectrum is absorbed efficiently. Specially, the energy content in the infrared region of the solar spectrum is significant which gets absorbed poorly in ultra-thin c-Si solar cell and mostly transmitted through the material. The most straightforward solution to this problem is to employ light trapping. This process involves “folding” the light multiple times into the absorbing region of the solar cell, thereby increasing the optical path length of the light and hence the probability of its absorption inside the solar cell. In other words, light trapping essentially increases the “optical” thickness of the absorber material. The ratio between the optical thickness and the absorber physical thickness is known as optical path length factor and is an important parameter in comparing different light trapping schemes.

Most commonly, light trapping is done by modifying the surface of the solar cell to increase the probability of total internal reflection. Also, it involves using anti-reflection coating in order to reduce the reflectivity at the air/absorber interface by using a transparent material allowing gradual change in the index of refraction. By doing so, the reflection of the light is reduced and it gets reflected back into the active volume several times before leaving the structure. There have been considerable efforts from the research community to address this issue by designing different schemes of light trapping. Most common technique is to texture the Si surface either to form upright or inverted regular pyramids^9–11^ or by random textures^12^. The texturing is mostly suitable for bulk Si solar cells. For ultra-thin solar cells having absorber thickness below 10 *μ*m, there are other techniques like - (i) nanophotonic light trapping that includes periodic semiconductor or dielectric structures^4^ e.g. one dimensional (1D), single period or dual period two dimensional (2D) diffraction gratings, photonic crystals^13^, nano-cones^14,15^ etc., (ii) plasmonic structures^16,17^, (iii) randomly structured semiconductor surfaces^18^, (iv) Si nanowires^19^ etc. Also, there are techniques which do not require physical nano-structuring but effectively increase the path length by increasing the local density of states^20^ or introducing frequency shift^21^.

The more light is trapped in an ultra-thin c-Si solar cell, the more confined the photogenerated carriers get inside the solar cell. This increases the minority carrier concentration at the metal contact interface. Given that the absorber lifetime remains high, with absorber thickness scaling, the contact recombination becomes a dominant component in the overall loss mechanism. In this paper, the problem of imperfect contact is addressed for ultra-thin c-Si solar cell. Specifically, our simulation study shows that contact selectivity problem is more prominent when light trapping is employed to increase the light absorption. Improving the contact selectivity can improve the performance more for the case where light trapping is involved compared to the case of planar Si cell without any light trapping.

With respect to photovoltaics, contact selectivity is defined as the ability of a contact to pass one type of carrier (electrons/holes) while blocking the other type (holes/electrons). The contact which allows electrons to pass and blocks holes is called electron selective contact (ESC)/hole blocking contact (HBC). The other type of contact that allows holes to pass and blocks electrons is called hole selective contact (HSC)/electron blocking contact (EBC).

**Figure 1:**
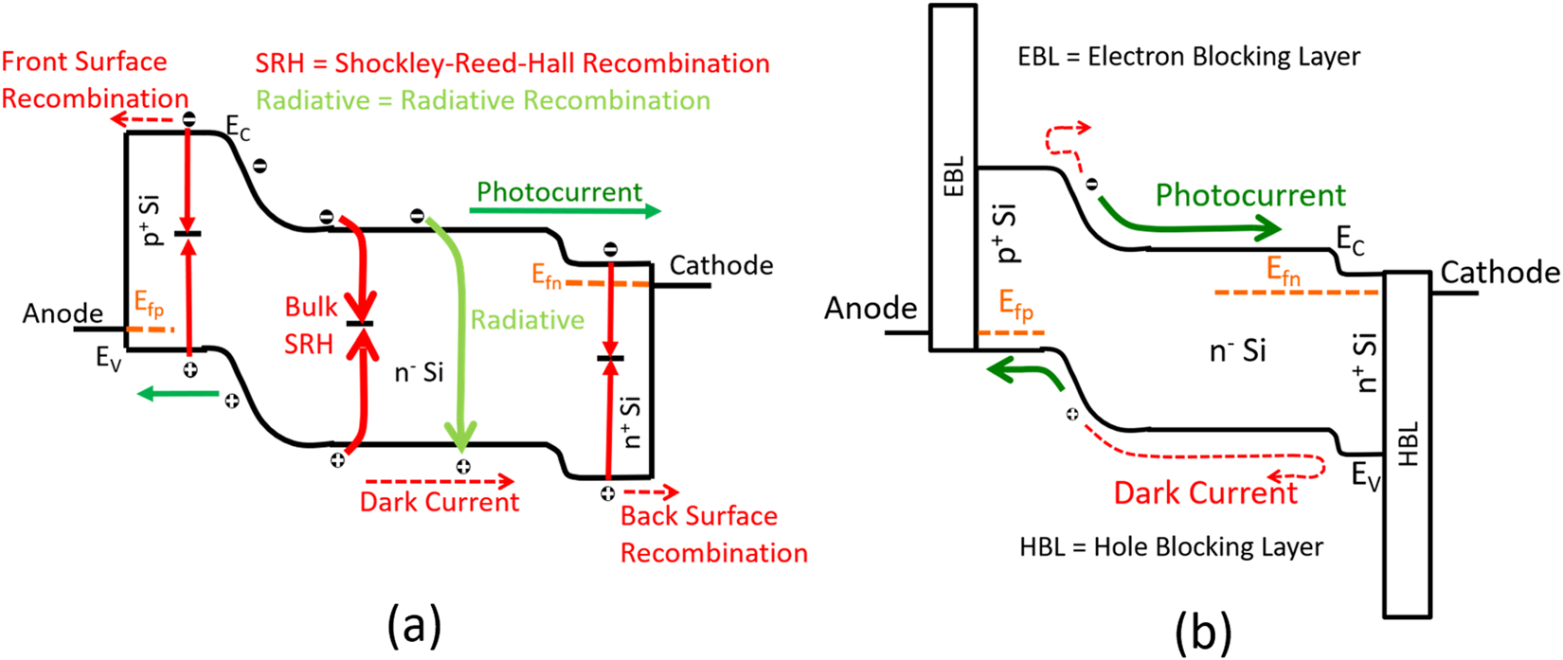
Energy band diagram of a (**a**) p^+^/n^−^/n^+^ junction solar cell showing the common recombination mechanisms, (**b**) solar cell with band engineered layer enhancing contact selectivity. EBL = Electron Blocking Layer and HBL = Hole Blocking Layer.

Typically, a p^+^/n^−^/n^+^ or n^+^/p^−^/p^+^ homojunction fabricated by doping c-Si can perform the basic photovoltaic process. The lightly doped middle layer (n^−^/p^−^) is the thickest region of the cell mostly absorbs the light and generates electron-hole pairs (ehp). The contact regions are highly doped junctions, whose thicknesses are determined by the requirement for lateral conduction and contact resistivity. Both n^+^ and p^+^ contacts ensure that each contact can pass only one type of carrier while blocking the other type. The blocking function of the contacts requires the band bending between the differently doped junctions. In the regions close to the junction there is high electric field, which creates the force to separate ehp. In between the junction region lies the quasi-neutral region where the dominant method of transport is through diffusion. Figure 1a shows the energy band diagram of a p^+^/n^−^/n^+^ junction solar cells with the common recombination mechanisms.

Since, the surface recombination velocity depends mostly on the interface trap states which does not scale with the thickness of the device, we can conclude that for an ultra-thin c-Si solar cell, lowering the surface recombination velocity can also improve the performance of the cell significantly. For ultra-thin c-Si solar cell contact becomes a bottleneck for higher performance. In other words, the contacts need to be more selective than what is obtained from direct metal contact to p^+^/n^−^/n^+^ or n^+^/p^−^/p^+^ homojunctions.

In order to evaluate the relative improvement from increasing the light trapping in ultra-thin c-Si solar cell, the thermodynamic limit of light trapping is a useful metric. This is known as Yablonovitch limit which gives an upper limit to light trapping. According to the limit, the internal light intensity can be enhanced by a factor of 2*n*^2^ from the incident light intensity where n is the refractive index of the media. For silicon, 2*n*^2^ ≈ 25. This means that a light ray in silicon is expected to make 25 passes on average before escaping^22^. Light trapping beyond the conventional thermodynamic limit has been demonstrated^20,21,23,24^.

In this paper, we show that using better photogenerated carrier management, performance enhancement is possible beyond the Yablonovitch limit. Improving contact selectivity is not light trapping in the usual sense. Since the ultimate goal of light trapping is to enhance the efficiency of the solar cell, increasing the contact selectivity is shown to be an effective technique to improve the performance of the solar cell beyond the thermodynamic limit of light trapping. Light trapping results in better photon management. Selective contact results in better carrier management. Our simulation study suggests that it is not one or the other but both that can ensure best possible energy harvesting from ultra-thin c-Si solar cell.

## Improving Contact Selectivity using Transition Metal Oxides

Figure 1b shows the energy band diagram of a contact structure to improve the contact selectivity beyond the metal/semiconductor contact. Here, ultra-thin (nanometer scale) selective contact interlayer is used between metal and silicon (shown as EBL and HBL) to block the dark current and allow the photocurrent^25^. EBLs have high conduction band offset and low valence band offset while the HBLs have high valence band offset and low conduction band offset with Si. The material can be either a semiconductor or an insulator. The basic rationale behind this contacting scheme is that although heterojunction between two semiconductors or semiconductor/insulator is far from ideal containing trap states to allow contact recombination current, the number of trap states is still a few orders of magnitude lower compared to the metal/semiconductor interface. As a result the contact recombination current can be reduced significantly allowing better carrier management. Also, due to high band offset for the minority carrier, the diode injection current in the forward bias will be completely blocked. The low band offset for the majority carriers will ensure that there is no significant barrier to the photo-generated current. Ideally the interface between EBL(HBL)/Si needs to be completely passivated. In practice, although perfect interface cannot be ensured, good interface passivation is possible using different surface treatment and annealing technique. Transition metal oxides (TMO) can be good candidates as selective contact layer for solar cells. Among different properties of TMOs, the most important ones that can be useful for this purpose are - (i) ability to be doped from defects or extrinsic dopant species, (ii) usually wide bandgap that is transparent to the visible spectrum of sunlight and (iii) widely varying band lineup with Si. The energy band alignment of different oxides and semiconductors with respect to Si and other semiconductors is shown in^25^. We can see that titanium oxide (TiO_2_) and zinc oxide (ZnO) have low conduction band offset, Δ*E*_*C*_, and high valence band offset, Δ*E*_*V*_ with respect to Si, making these suitable for electron selective contact or hole blocking layer (HBL). The opposite is true for nickel oxide (NiO) and copper aluminum oxide (CuAlO_2_), making these suitable for hole selective contact or electron blocking layer (EBL).

Moreover, TiO_2_ and ZnO are n type semiconductors, where both extrinsic and intrinsic doping through oxygen vacancy is possible^26^. ZnO has been shown to dope heavily with aluminum. On the other hand, NiO and CuAlO_2_ are p type semiconductors, where both extrinsic and intrinsic doping through cation vacancy is possible. All of these four materials have been studied as window layer or transparent conducting oxide (TCO) for photovoltaics^27–30^ due to their optical transparency in the visible spectrum and distinct semiconducting properties. In this paper, the simulation results shown are done using TiO_2_ and NiO to understand the effect of absorber thickness on contact selectivity. Evaluation and relative comparison of these materials from selective contact point of view was presented elsewhere^25^.

## Simulation Framework

**Figure 2:**
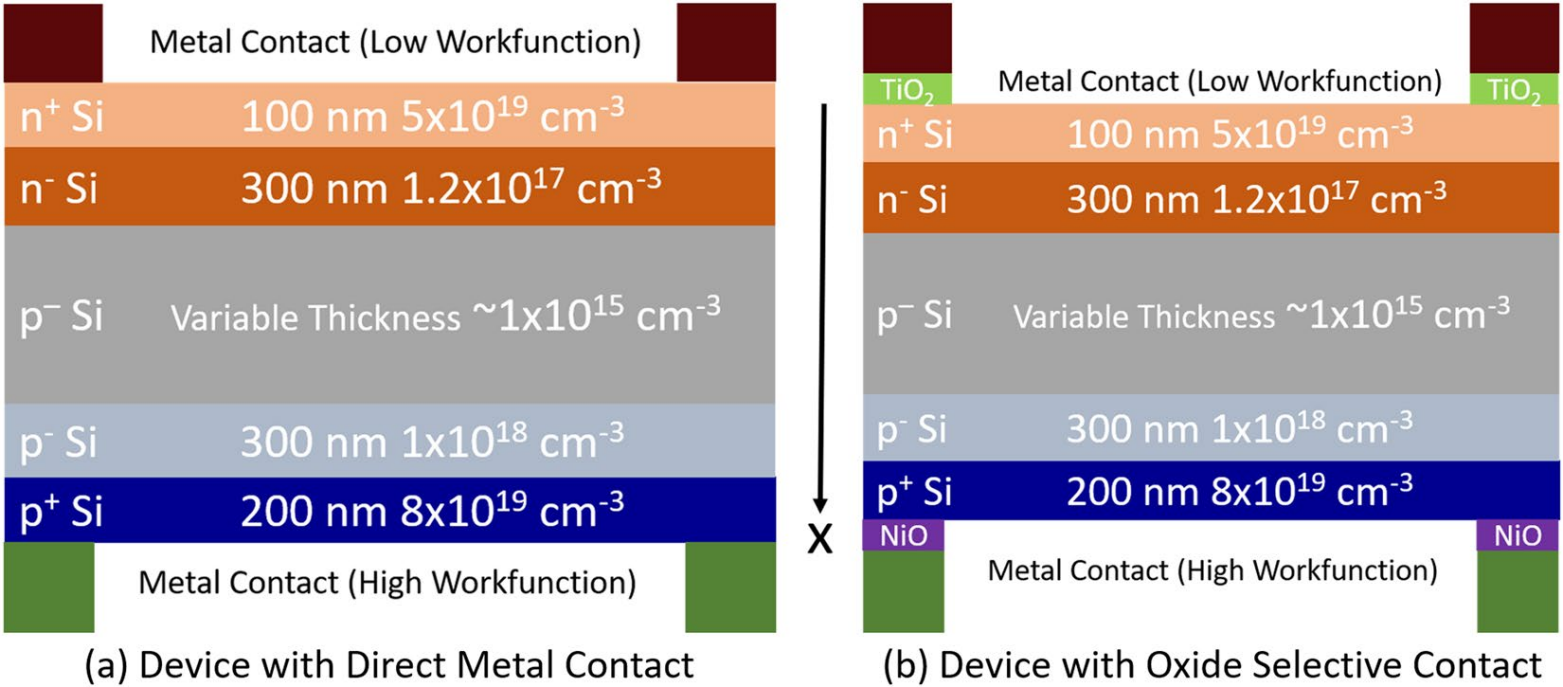
Schematic of the simulated device structures. (**a**) Control sample (**b**) Test sample.

Figure 2 shows the schematic of the device structure simulated in this work. The software package AFORS-HET 2.5 (Automat FOR Simulation of Heterostructures) is utilized in order to simulate the selective contact solar cells^31^. In AFORS-HET, The 1-D Poisson equation and the carrier continuity equations are solved within the optical carrier generation determined by the Beer-Lambert law as given below:

*Poisson Equation*,

1 $$\frac{{\varepsilon }_{0}{\varepsilon }_{r}}{q}\frac{{\partial }^{2}\varphi (x,t)}{\partial {x}^{2}}=p(x,t)-n(x,t)+{N}_{D}(x)-{N}_{A}(x)+\sum _{trap}\,{\rho }_{trap}(x,t)$$

*Transport Continuity Equation*,

2a $$-\,\frac{1}{q}\frac{{\partial }^{2}{j}_{n}(x,t)}{\partial {x}^{2}}={G}_{n}(x,t)-{R}_{n}(x,t)-\frac{\partial n(x,t)}{\partial t}$$

2b $$-\,\frac{1}{q}\frac{{\partial }^{2}{j}_{p}(x,t)}{\partial {x}^{2}}={G}_{p}(x,t)-{R}_{p}(x,t)-\frac{\partial p(x,t)}{\partial t}$$

*Beer-Lambert Law*,

3a $$G(x,t)={\int }_{{\lambda }_{{\min }}}^{{\lambda }_{{\max }}}\,d\lambda {{\rm{\Phi }}}_{0}(\lambda ,t)R(\lambda )A(\lambda ){\alpha }_{x}(\lambda ){e}^{-\frac{{\alpha }_{x}(\lambda )}{{\cos }(\gamma )}}$$

3b $${\alpha }_{x}(\lambda )=\frac{4\pi k(\lambda )}{\lambda }$$

Here, the meaning of the symbols are given below:

*ε*_*r*_ = Relative permittivity of the semiconductor; *ϕ* = Electric potential; *n*, *N*_*D*_ = Electron concentration, Donor doping concentration; *p*, *N*_*A*_ = Hole concentration, Acceptor doping concentration; *j*_*n*_/*j*_*p*_ = Electron/Hole current density; *G*_*n*_/*G*_*p*_ = Electron/Hole generation rate; *R*_*n*_/*R*_*p*_ = Electron/Hole generation rate; Φ_0_ = Incoming spectral photon flux; *R*(*λ*) = Reflectance of the contact; *A*(*λ*) = Absorptance of the contact; *α*_*x*_(*λ*) = Spectral absorption coefficient of the semiconductor; *k*(*λ*) = Extinction coefficient of the semiconductor; *cos*(*γ*) = Factor to consider path length enhancement of light from textured surface.

The absorber thickness is varied from 1 *μ*m to 120 *μ*m while keeping the high doped region same. We only scale the absorber thickness because the need to ensure low series resistance of the cell restricts the minimum thickness of the emitter and back surface field regions. The standard AM1.5 G spectrum is used to calculate photo-generated carriers given the experimentally extracted absorption coefficient of Si. The oxides were simulated as wide bandgap semiconductors with the parameters taken from the published literature and are assumed to not absorb any photons. The oxide properties used in this simulation study is given in Table 1.

**Table 1:** Material Parameters of the Oxides^25–27,32^.

Metal Oxide	TiO_2_	NiO
Dielectric Constant, k	86	11.75
Electron Affinity, *χ* (eV)	4	1.8
Bandgap, E_g_ (eV)	3.2	3.6
Effective Density of States (Conduction Band), N_C_ (cm^−3^)	7.93 × 10^20^	8.87 × 10^18^
Effective Density of States (Valence Band), N_V_ (cm^−3^)	1.79 × 10^19^	7.57 × 10^18^
Electron Mobility, *μ*_*n*_ (cm^2^/*V*_*s*_)	0.1	0.1
Hole Mobility, *μ*_*p*_ (cm^2^/*V*_*s*_)	0.1	0.1
*ϕ*_*CNL*_ (w.r.t. Vacuum) (eV)	4.6	4.85
Pinning Factor, S	0.24	0.26

**Figure 3:**
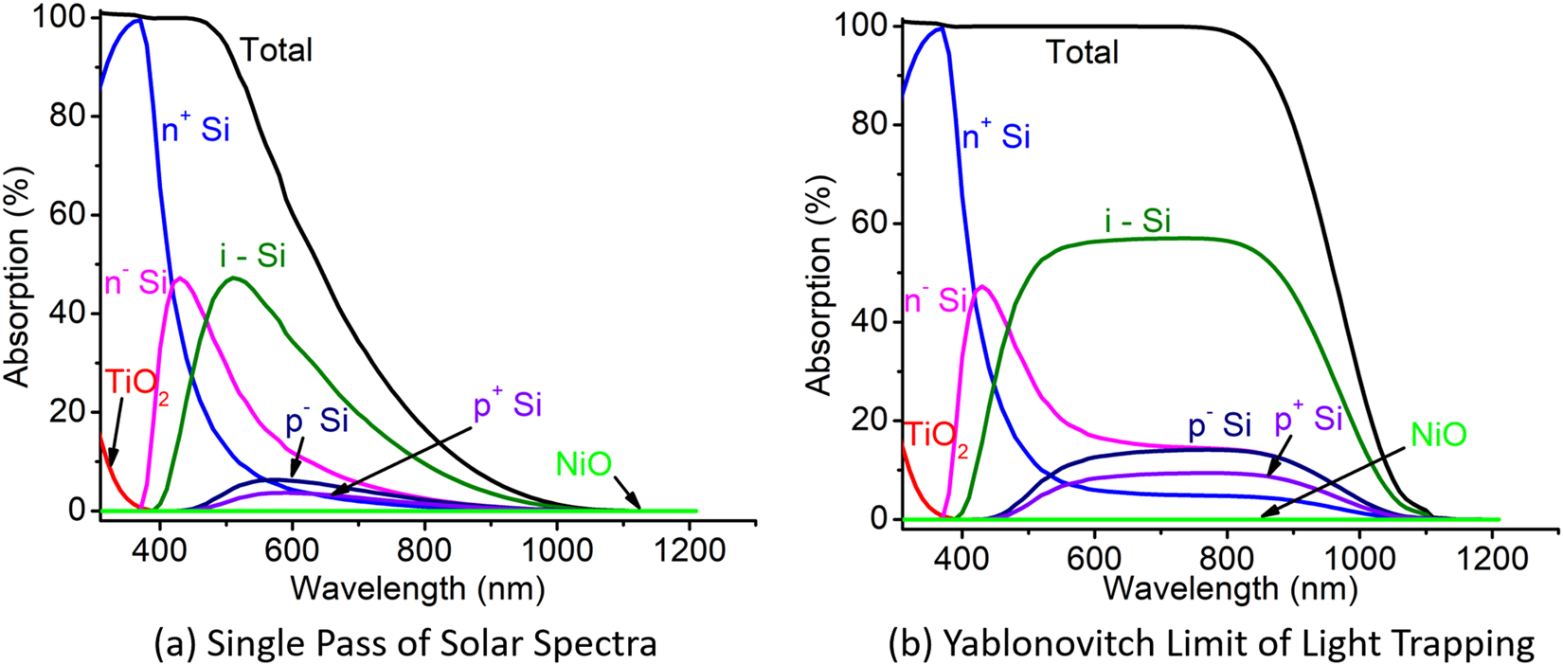
Absorption spectra of different layers in a thin c-Si solar cell for the cases of (**a**) single light pass and (**b**) Yablonovitch limit of light trapping.

For the purpose of light trapping simulation, multiple passing of incident light is considered, which in each pass loses its intensity as determined by the Beer-Lambert law. Since, Yablonovitch limit stipulates an average of 25 passes in silicon before the light escapes, we consider 25 passes of light to be equivalent of simulating the thermodynamic limit of light trapping. The absorption spectra for different layers of the device shown in Fig. 2b is shown in Fig. 3. We observe that with single pass in a thin c-Si solar cell a significant portion of infrared is lost. Light trapping enhances the effective path length which improves the absorption in the infrared region of the spectra.

We assume a constant defect distribution along the bandgap of Si at the oxide/Si interface according to the following equation,

4 $${N}_{trap}(E)=({E}_{trap}^{end}-{E}_{trap}^{start}){N}_{trap}^{const}\vartheta (E-{E}_{trap}^{end})\vartheta ({E}_{trap}^{start}-E)$$

Here, ${E}_{trap}^{start}$ and ${E}_{trap}^{end}$ denotes the start and end energy of the energy interval within the bandgap where a constant defect density is assumed, ${N}_{trap}^{const}$ denotes the constant defect density per energy and *ϑ*(*E*) is the step function.

**Figure 4:**
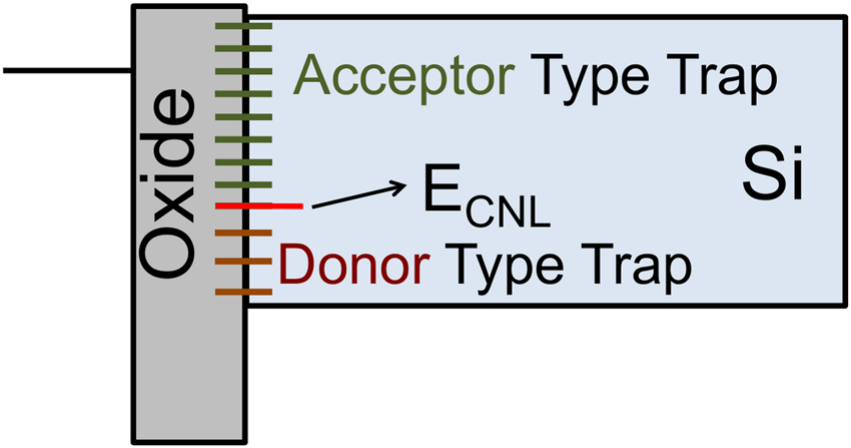
Schematic of the interface defect distribution considered in this work.

The character of the defects switches from donor type to acceptor type at the E_CNL_ of Si as we move from the valence band towards the conduction band (Fig. 4). In this work, we assumed ${N}_{trap}^{total}$ to be 10^10^ cm^−2^. Although it is an optimistic assumption, this gives a comparative picture of how much benefit can be obtained from band offset asymmetry for different absorber thicknesses. Compared to direct metal contact, oxide/Si interface always has orders of magnitude lower trap density, so in all practical purpose the conclusions about the effectiveness of oxide selective contacts are valid. The surface lifetime (*τ*_*s*_) is related to the defect density as,

5 $${\tau }_{s,n/p}=\frac{1}{{\sigma }_{n/p}{\upsilon }_{th}{N}_{trap}^{total}}$$

Here, *σ*_*n*/*p*_ corresponds to the capture cross-section of electrons (n) or holes (p), *υ*_*th*_ is the thermal velocity of the carriers. This corresponds to the surface lifetime (*τ*_*s*_) of 1 ms at the metal oxide/Si interface assuming electron/hole capture cross section of the defect is 10^−14^ cm^2^. The effective lifetime (*τ*_*eff*_) is related to the bulk lifetime (*τ*_*b*_) and the surface lifetime (*τ*_*s*_) as,

6 $$\frac{1}{{\tau }_{eff}}=\frac{1}{{\tau }_{b}}+\frac{1}{{\tau }_{s}}$$

*τ*_*b*_ is considered to be either 10 ms and 1 *μ*s, where the effective lifetime is mostly dominated by *τ*_*s*_ and *τ*_*b*_ respectively. Constant defect density is a simplified picture. However for qualitative comparison, this is sufficient.

## Results and Discussion

### Oxide Selective Contact for the Ideal Cell

**Figure 5:**
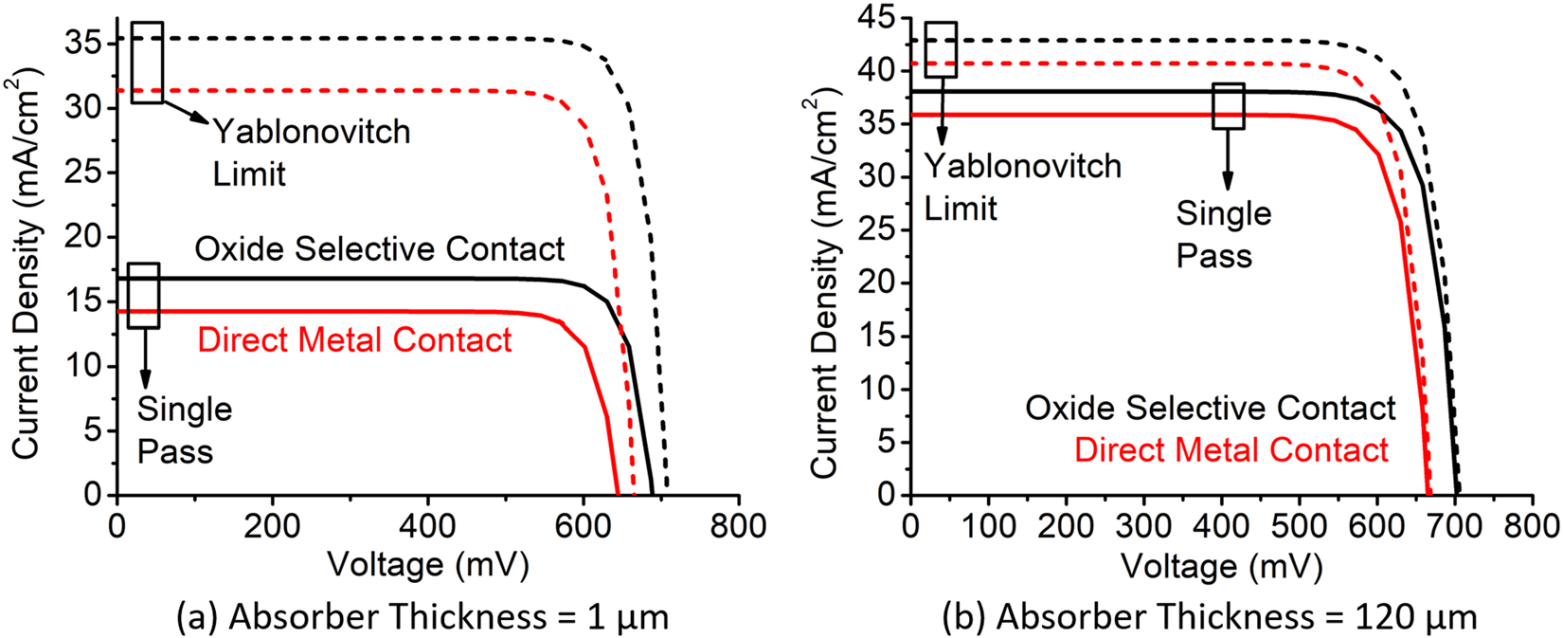
Comparison of photogenerated current-voltage characteristics of the cell at AM 1.5 G spectra between direct metal contact and oxide selective contact for two terminal cases of absorber thickness.

In this section, we present the simulation results for the base condition of the cell where the doping of the c-Si absorber layer is 1 × 10^15^ cm^−3^ and the bulk lifetime is 10 ms. These values represent the typical scenario of a high quality c-Si solar cell (either a float-zone wafer or epitaxial growth). The absorber thickness is varied from 1 *μ*m to 120 *μ*m. Figure 5 compares the photogenerated J-V characteristics of the cell for the two terminal cases of absorber thickness. In each figure, the J-V curves are compared between direct metal contact and oxide selective contact (both EBL and HBL). For each type of devices, two terminal cases of light trapping schemes are shown. One case is single pass which simulates the device assuming only one pass of solar spectrum through the device. The other case is “Yablonovitch” limit which simulates the device assuming 25 passes of solar spectrum through the device. This figure demonstrates the benefit of incorporating oxide selective contacts clearly. Extracted from the J-V curves are the performance metrics - V_*oc*_ and J_*sc*_.

**Figure 6:**
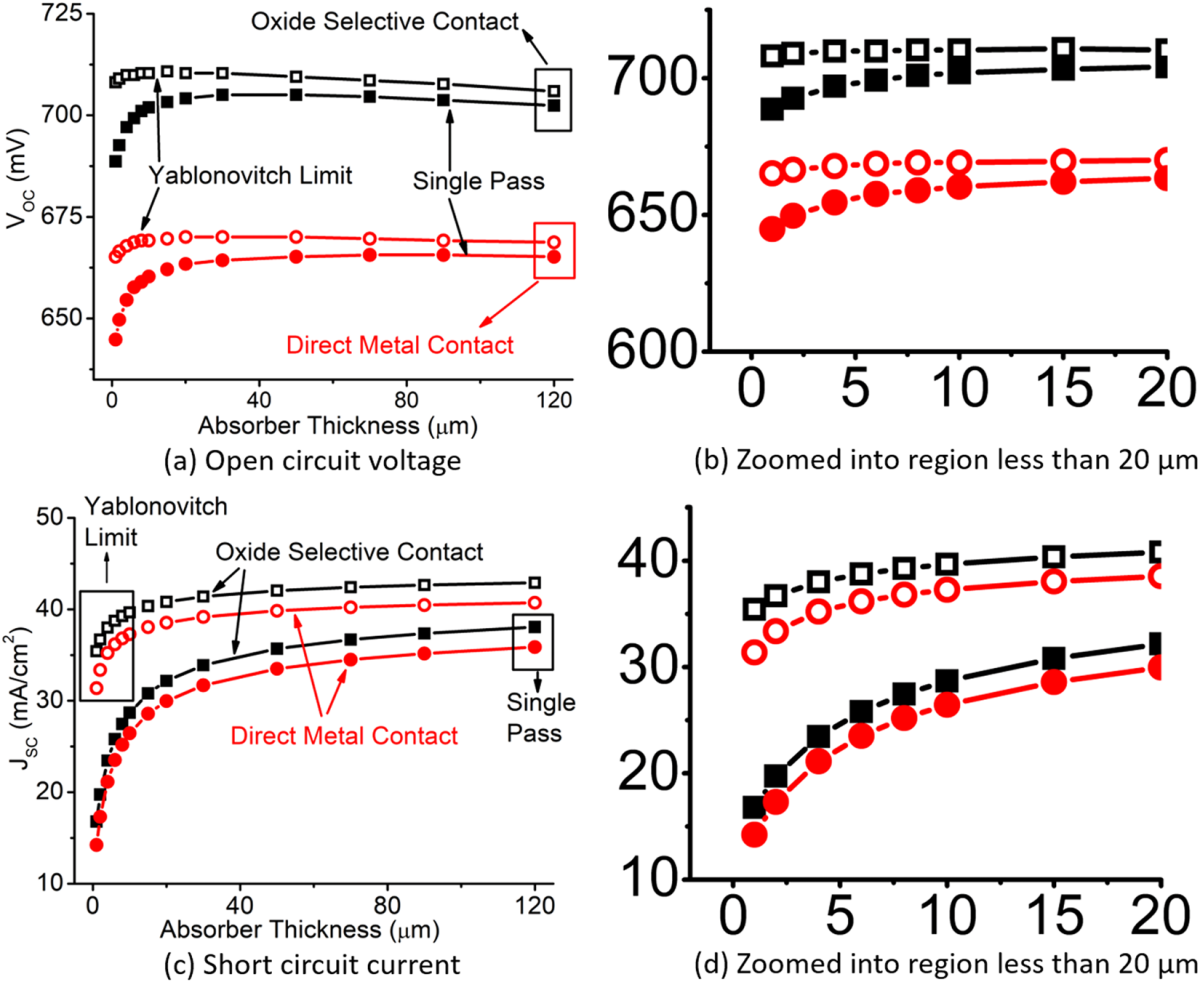
(**a**,**b**) Open circuit voltage and (**c**,**d**) short circuit current of thin c-Si solar cell compared between direct metal contact and oxide selective contact as a function of absorber thickness.

Figure 6 shows these performance metrics (V_*oc*_ and J_*sc*_) of c-Si solar cells shown in Fig. 2 as a function of absorber thickness. We observe dramatic change in V_*oc*_ and J_*sc*_ below 20 *μ*m absorber thickness. A zoomed-in version of the Fig. 6a,c is shown to the right of the corresponding figure (Fig. 6b,d respectively) to observe the x-axis range below 20 *μ*m more clearly.

We observe that V_*oc*_ increases slightly as the thickness decreases from 120 *μ*m. However, below 20 *μ*m the V_*oc*_ starts decreasing significantly. Integrating oxide selective contact can improve open circuit voltage significantly, although the shape of the curve remains same. For thicknesses below 20 *μ*m the forward injection current over the barrier becomes significantly higher, which is responsible for the sharp decrease in V_*oc*_. By blocking the forward injection current, oxide selective contacts can reduce the dark current that results in an increased V_*oc*_. Note that the improvement in V_*oc*_ from the oxide selective contact exceeds beyond that is obtained from light trapping.

**Figure 7:**
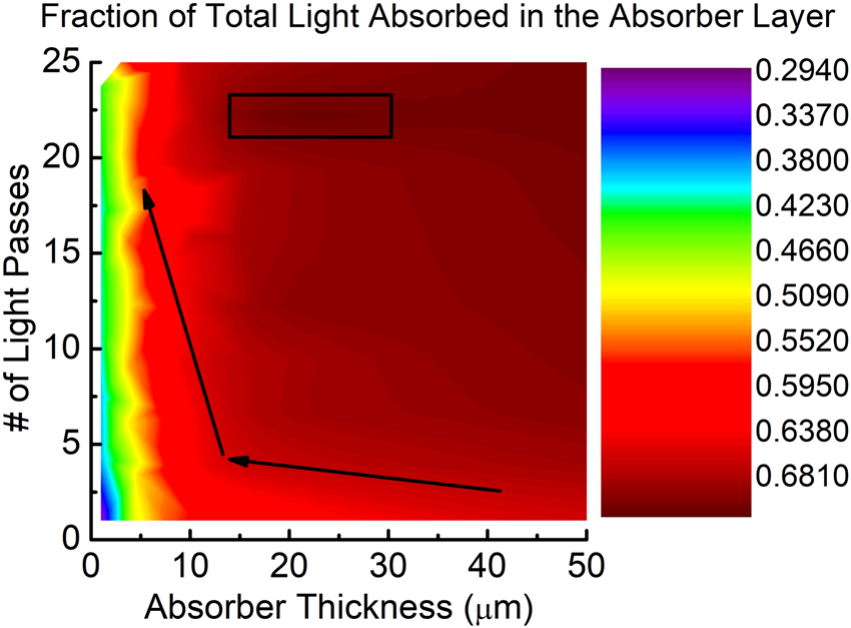
Fraction of the total solar spectra (AM1.5 G) absorbed in the absorber layer of a solar cell as a function of absorber thickness and number of light passes through the cell.

J_*sc*_ follows a different trend than V_*oc*_ as a function of absorber thickness. Without light trapping, J_*sc*_ starts decreasing fast when the absorber thickness is reduced. J_*sc*_ directly depends on the amount of light absorbed by the cell. So a significant improvement comes from increasing the light trapping. However, the benefit of integrating oxide selective contact is also visible in J_*sc*_. The amount of J_*sc*_ increase is more in light trapped case compared to the case when there is no light trapping. Specifically, when the curve is zoomed below 20 *μ*m (Fig. 6d), we observe the clear advantage of adding oxide selective contact to the ultra-thin solar cell having light trapping. This is because light trapping increases the carrier concentration in a thinner semiconductor film which brings more minority carriers close to the contacts. This carrier confinement due to light trapping in ultra-thin c-Si solar cells can be observed in Fig. 7. In this figure, the color contour represents the fraction of the total light absorbed in the variable thickness absorber layer (Fig. 2). The lower this fraction is, the more carriers are generated in the highly doped contact region which is subject to the surface recombination. The edge of the contours are shown with the arrow. It can be observed that when thickness decreases, the fraction decreases slightly. Roughly 20 *μ*m thickness is when the fraction reaches its peak (shown with the black rectangle). It means that below this thickness, more light trapping will bring more minority carriers near the surface region of the cell which increases the probability of surface recombination.

**Figure 8:**
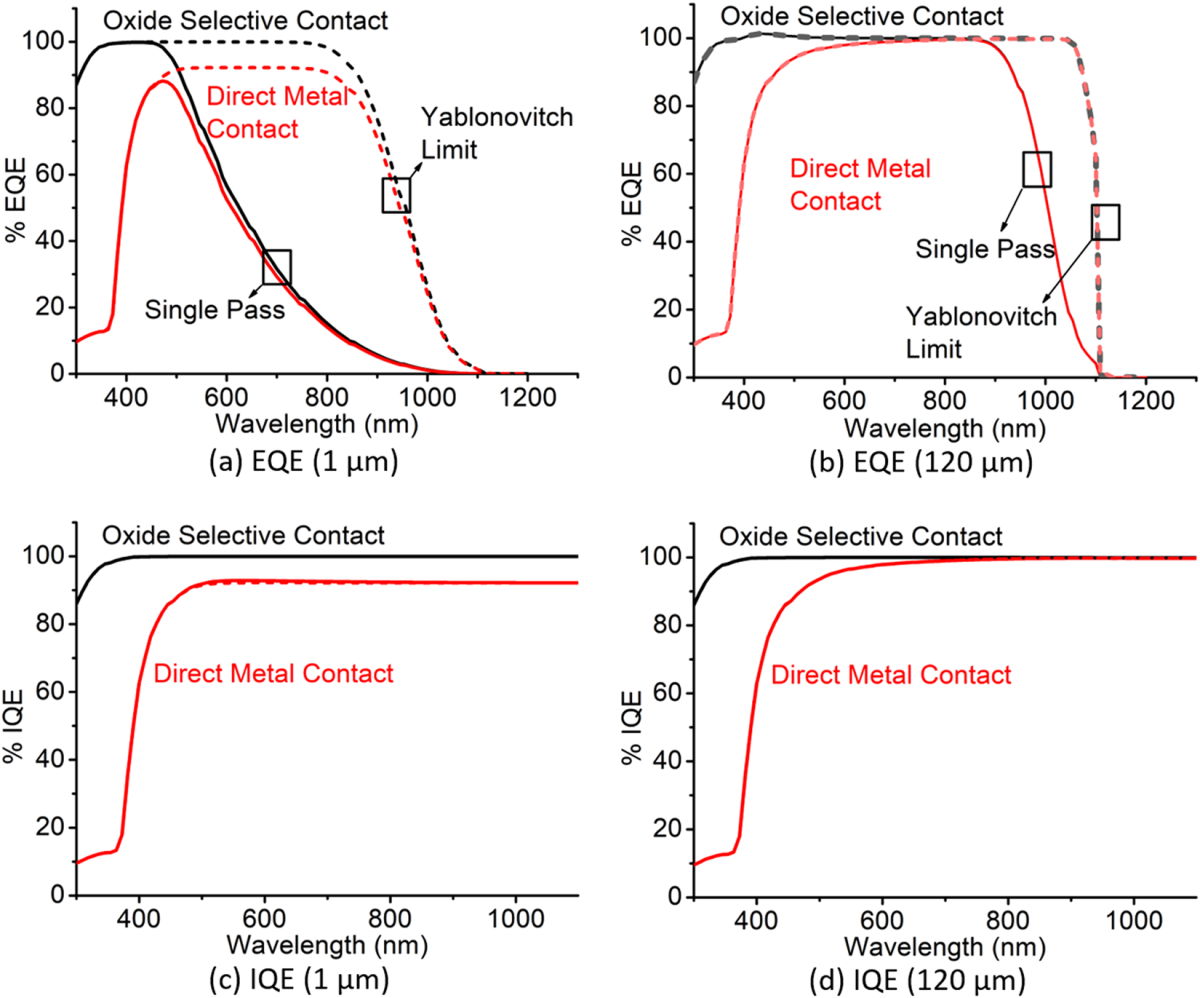
External Quantum Efficiency (EQE) and Internal Quantum Efficiency (IQE) of (**a**,**c**) 1 *μ*m and (**b**,**d**) 120 *μ*m absorber thickness solar cell compared between direct metal contact and oxide selective contact for different light trapping conditions.

Irrespective of the light trapping, oxide selective contacts improve J_*sc*_ because it increases the collection efficiency of the near-UV (higher energy) photons which are typically absorbed near the interface. Figure 8 shows the external quantum efficiency (EQE) and the internal quantum efficiency of two terminal cases of absorber thickness comparing the direct metal contact with the oxide selective contact. IQE (Fig. 8c,d) is the fraction of the extracted carrier with respect to the absorbed photons, so it is independent of light trapping. EQE (Fig. 8a,b) is the fraction of the extracted carrier with respect to the total photons, so it depends on the reflection and transmission loss. From both EQE and IQE, it is clear that the higher energy photons are collected efficiently, something which cannot be improved by light trapping only due to surface recombination. Oxide selective contacts increases the carrier collection from the photons of this part of the spectra. This also suggests that due to non-ideal interface passivation, quantum efficiency of near-UV photons will be impacted. At the extreme condition, it will be equal to the direct metal contact. Typically interface trap density is lower in oxide/Si interface compared to the metal/Si interface which justifies the use of oxide selective contact for thin c-Si solar cells.

**Figure 9:**
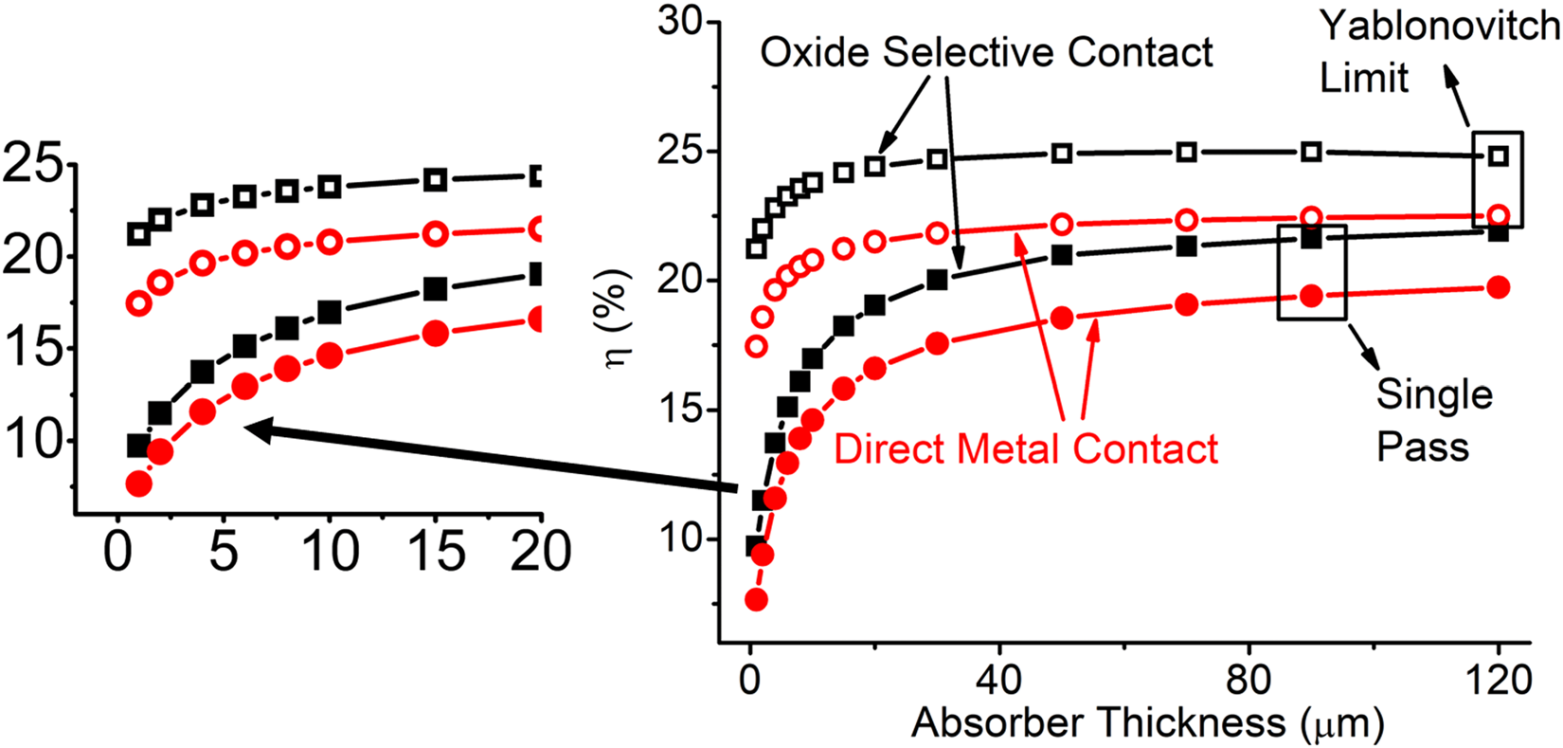
Efficiency of the c-Si solar cell as a function of absorber thickness showing comparisons between direct metal contact and oxide selective contact devices for single pass and thermodynamic light trapping case.

Finally, the efficiency is shown in Fig. 9 comparing direct metal contact with oxide selective contact as a function of absorber thickness. This roughly follows Fig. 6c in trends. However, the zoomed in version reveals that for absorber thickness below 20 *μ*m the benefit of incorporating oxide selective contact is significant. Specially, oxide selective contact improves the efficiency of ultra-thin cells beyond “Yablonovitch” limit of light trapping. Nevertheless, the improvement from the oxide selective contact without light trapping reaches close to the “Yablonovitch” limit near higher absorber thickness (>100 *μ*m). This is important because it can allow us to reap the same benefit of light trapping just by improving the contacts which is a relatively cheaper process than surface texturing.

**Figure 10:**
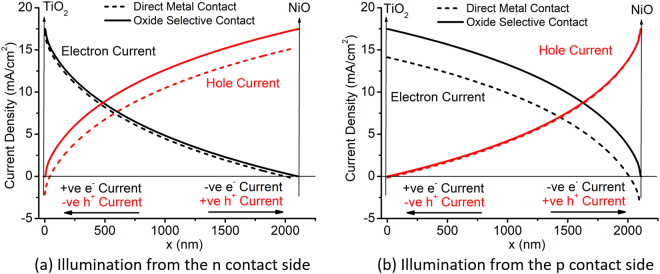
Current density inside the cell as a function of distance, x (shown with the arrow), in the short circuit condition for different direction of illumination.

In order to understand the mechanism of contact selectivity enhancement from device perspective, Fig. 10 is studied. In this figure, electron and hole currents are plotted separately as a function of position (x) in the cell from one contact to the other (increasing x means from top to bottom contact with respect to Fig. 2). The contact edge between oxide/Si is shown with the vertical line in each sub-figure. For direct metal contacted device this vertical line corresponds to the metal/Si interface. The two different sub-figures (10a and b) are shown for the illumination from n and p side respectively. The UV portion of the spectra is absorbed at a shallow depth from the illumination side. This means that for a single pass of light more photo-generated carriers are generated near the contacts where the illumination is from. By simulating two different sides of illumination, we can show the effect of electron and hole selective contacts separately. In Fig. 10a, we observe that at the TiO_2_/Si interface, the oxide suppresses (makes it zero at the interface) the hole current that is negative. Negative hole current corresponds to the holes traveling towards the TiO_2_/Si contact. This is the unwanted dark current that TiO_2_ blocks as an electron blocking layer. Also, the electron current is almost same at the interface which proves that TiO_2_ contact blocks one type of carrier (hole) and allows the other type (electron) - the definition of contact selectivity. Similarly, Fig. 10b shows that NiO blocks electron current which is negative meaning it was flowing in the +ve x-direction towards the p type contact. At the same time, the hole current remains almost the same proving its contact selectivity properties.

In practice, the contacts are not ideal. The suppression of the minority carrier is hampered from the interface recombination due to the trap states at the oxide/silicon hetero-interface. Sometimes, oxide can have small barrier to the majority carrier due to imperfect band alignment. Nevertheless, the basic principle of blocking the minority carrier from reaching metal/semiconductor interface remains same. These non-idealities bring the challenges of optimizing the contacts and choosing the right material. The detailed optimization of the contacts is presented elsewhere^25^. We have presented the comparison in performance for TiO_2_ and ZnO electron selective contact in the supplementary information (Fig. S1) to illustrate the similary of the selective contacts having similar band lineup in ideal condition.

### Oxide Selective Contact for Low Bulk Lifetime Absorber

**Figure 11:**
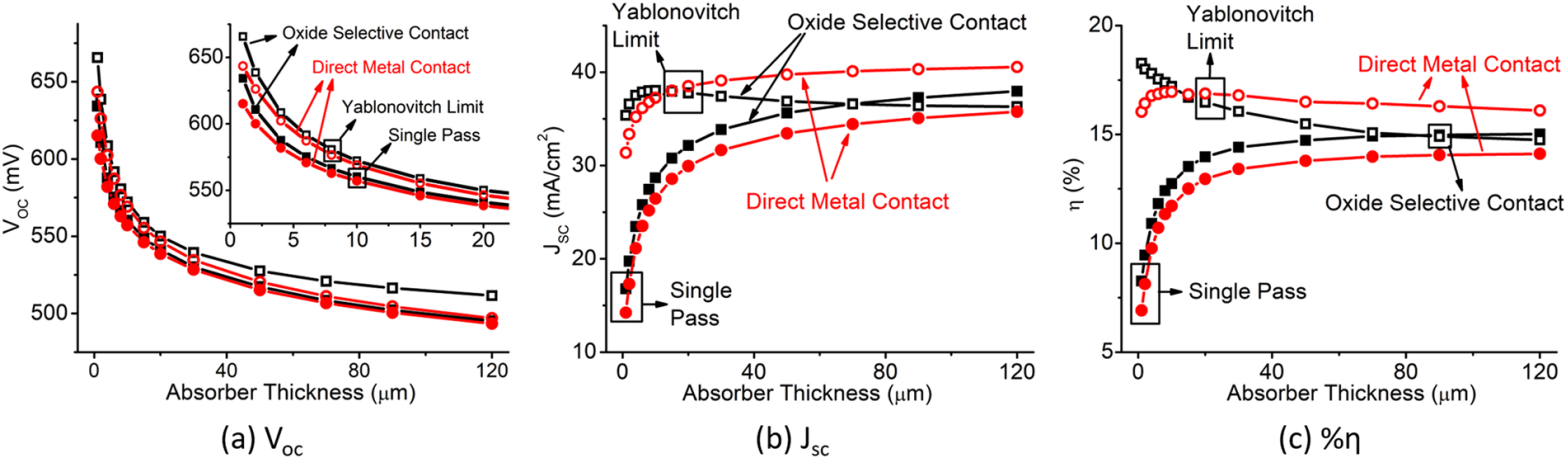
(**a**) Open circuit voltage, (**b**) short circuit current, and (**c**) efficiency of thin c-Si solar cell compared between direct metal contact and oxide selective contact as a function of absorber thickness considering the bulk lifetime of 1 *μ*s.

In the previous subsection, we have assumed high bulk lifetime for the absorber material, but the conclusions will hold for low-lifetime absorber materials as well. In case of low-lifetime materials, the contact selectivity impacts the performance based on the diffusion length of the carriers inside the material. When the thickness of the absorber material is close to the diffusion length, the cell performance is limited by contact selectivity and greater V_*oc*_ improvement can be seen by improving the selectivity when the cell is optically enhanced. That is why in Fig. 11, we observe a cross-over point around 15 *μ*m absorber thickness between direct metal contact and oxide selective contact. This is when it is clearly observed that oxide selective contact can provide gains in efficiency in addition to light trapping when the absorber thickness is scaled aggressively. For thicker absorber thickness, increasing light trapping brings diminishing returns from light trapping as the diffusion length is much smaller. This case is representative of solar cells having deposited poly-crystalline Si absorber on flexible substrate. Our simulation suggests that introducing oxide selective contact is an effective and cheaper way to increase efficiency for these cells.

### Oxide Selective Contact for Highly Doped Absorber

**Figure 12:**
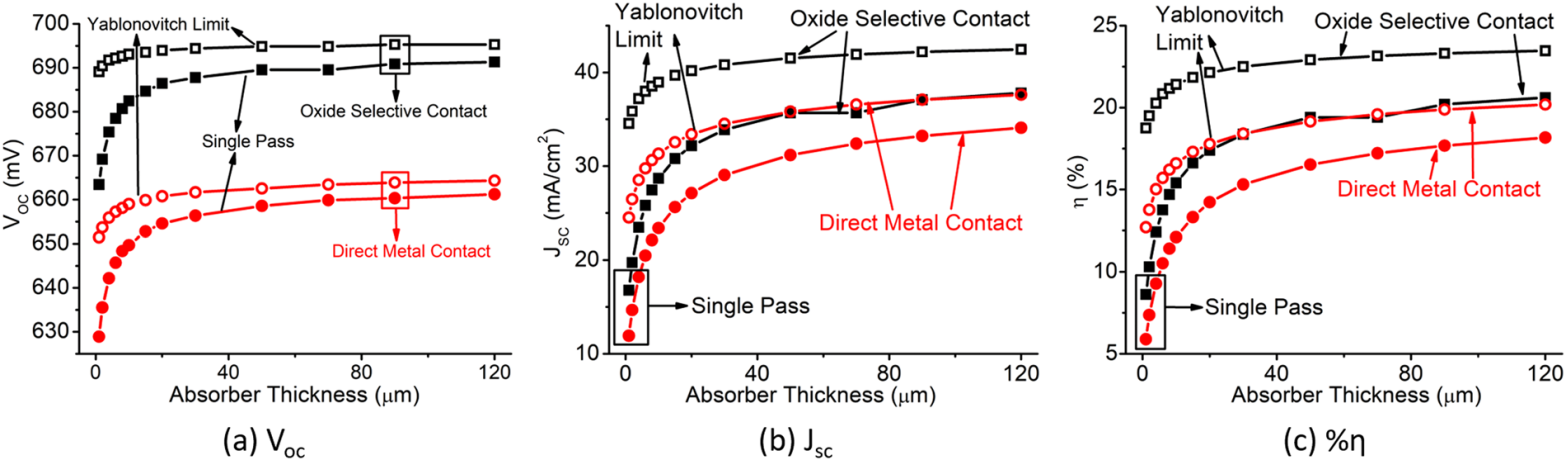
(**a**) Open circuit voltage, (**b**) short circuit current, and (**c**) efficiency of thin c-Si solar cell compared between direct metal contact and oxide selective contact as a function of absorber thickness considering high absorber doping 1 × 10^18^ cm^−3^.

In this section, we study the effect of absorber doping in solar cells having oxide selective contacts. Figure 12 shows the cell performances for the cell where the absorber doping is 1 × 10^18^ cm^−3^. Although, typically absorber doping is much lower than this in a practical solar cell, this analysis is useful for the better understanding of oxide selective contacts. The trend of the curves is not significantly different from the low-doping case (Figs 6 and 9). However, for thicker absorber the efficiency with oxide selective contact is comparable to the cell with direct metal contact and enhanced by only light trapping. The quasi-neutral region of highly doped absorber is larger compared to the lightly doped absorber. Minority carrier diffusion is higher which increases the dark current. Oxide selective contact decreases the dark current by blocking one type of carrier in each contact. Most importantly, we observe significant efficiency drop for ultra-thin solar cell. Oxide selective contact improves it significantly which illustrates the effectiveness of oxide selective contacts in a wide range of design space.

## Conclusion

This paper considers the two performance limiting factors of ultra-thin c-Si solar cell and compares the relative importance of addressing both of the issues. The important finding from this work is that carrier selectivity improvement becomes more important when light trapping is employed to increase the light absorption. This brings more carriers close to the contact, which results in increasing probability of contact recombination. Light absorption enhancement provides superior light management while contact selectivity improvement provides superior photo-generated carrier management. This work proves that both of the techniques are necessary to push the performance of the solar cell towards theoretical limit. The question of designing a better light trapping versus improving the contact selectivity becomes more significant as we scale the Si absorber thickness below 20 *μ*m. This limit is obtained for silicon, but the conclusions are equally valid for other thin film solar cell technology.

### Data availability

The datasets generated during and/or analysed during the current study are available from the corresponding author on reasonable request.

## Electronic supplementary material

Supplementary Figure
