# Supplementary material for: Limitation of Optical Enhancement in Ultra-thin Solar Cells Imposed by Contact Selectivity
Source: Sci Rep. 2018 Jun 11;8:8863. doi: 10.1038/s41598-018-27155-0 (PMC5995827; doi:10.1038/s41598-018-27155-0)
Supplement: Supplementary file 1 — Supplementary Figure [file 41598_2018_27155_MOESM1_ESM.docx]

**Supplementary Information for:**

**Limitation of Optical Enhancement in Ultra-thin Solar Cells Imposed by Contact Selectivity**

Raisul Islam^1^* and Krishna Saraswat^1^

*^1^Department of Electrical Engineering, Stanford University, 420 Via Palou Mall, Stanford, CA 94305, USA*

*Corresponding author, email: [raisul@stanford.edu](mailto:raisul@stanford.edu) (R. Islam).


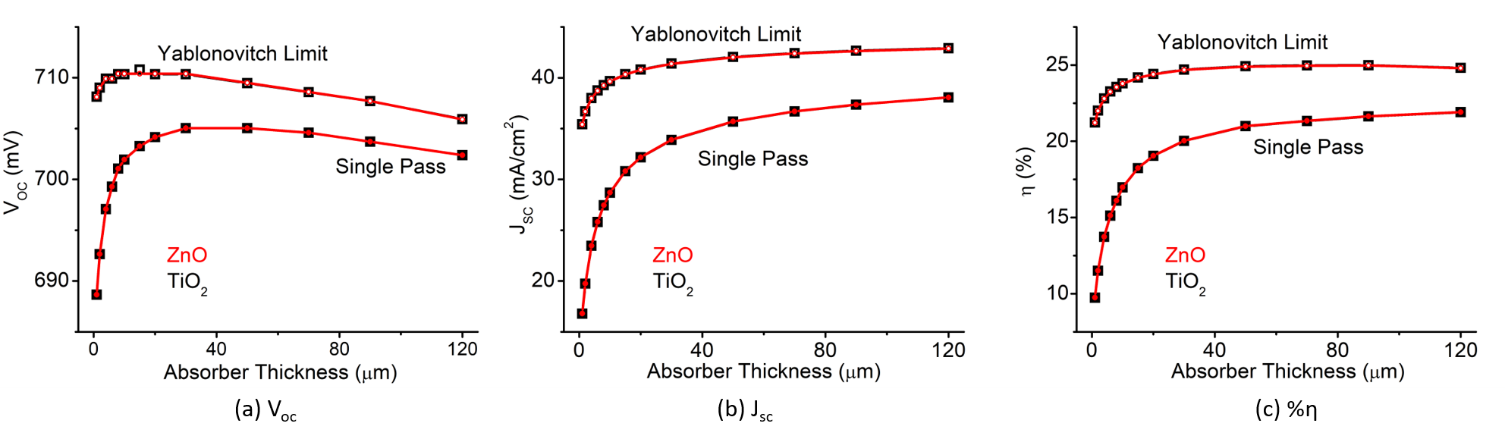


**Figure S1.** (a) Open circuit voltage, (b) short circuit current, and (c) efficiency of thin c-Si solar cell compared between direct metal contact and oxide selective contact as a function of absorber thickness considering ZnO (red) and TiO_2_ (black) as electron selective contacts. NiO is kept as hole selective contacts for both the cases.
